# Does Resilience Help in Reducing Burnout Symptoms Among Chinese Students? A Meta-Analysis

**DOI:** 10.3389/fpsyg.2021.707792

**Published:** 2021-08-17

**Authors:** Zhun Gong, Chunqin Li, Xinian Jiao, Qunzhen Qu

**Affiliations:** ^1^Department of Psychology, Normal College, Qingdao University, Qingdao, China; ^2^School of Economics and Management, Shanghai Maritime University, Shanghai, China

**Keywords:** student burnout, resilience, gender difference, decline effect, gray literature bias

## Abstract

As society has evolved, student burnout has become a common problem in schools around the world, including in China. Therefore, the purpose of the current study is to explore whether resilience is related to student burnout in China and to examine the changing trend of resilience and student burnout. Moreover, we will assess gender differences and possible biases, including publication biases, small-study biases, gray literature biases, and decline effects. This meta-analysis included 34 studies, with a total of 81 effect sizes and a total sample size of 22,474. We found that resilience was negatively correlated with student burnout in the Chinese context. We also found evidence of gray literature bias in student burnout, which needs to be verified by subsequent studies. However, we found that there were decline effects in resilience, possibly because, as culture evolves, people become more focused on themselves; thus, their collective behaviors decline, leading to a decrease in their ability to adapt to the collective and the environment. We also found similar decline effects at the individual level; that is, resilience might decrease with individual age stages (from the primary to college stage), which might be related to the use of immature defense mechanisms against stress by students.

## Introduction

### Student Burnout

The term student burnout came from job burnout; in the 1980's, a phenomenon similar to job burnout started surfacing among students and attracted widespread attention. Pines et al. (1981) proposed the term “student burnout” in their study, and they thought that student burnout was a negative reaction that occurred when students were under long-term academic pressure and heavy burdens. Since then, scholars around the world have carried out a series of studies on student burnout. Maslach (1982) divided burnout into three stages. First, job pressure exceeds the range that individuals can bear. Second, individuals draw a clear boundary from the job with attitudes of alienation and unwillingness to expose themselves. Third, individuals are aware that there is a huge gap between actual performance and expectations, which leads to low personal accomplishment. Therefore, Maslach (1982) considered burnout to be an attitude of alienation, sarcasm, or denial, and a combination of exhaustion, deindividualization, and self-negation. Based on this, Maslach and Jackson (1986) proposed the three-factor model of job burnout, which included exhaustion, cynicism, and feelings of inadequacy. Subsequently, the terms of the three-factor model were improved to exhaustion, cynicism, and inefficacy (Schaufeli et al., 1996), further stating that burnout had stage characteristics. At present, the three-factor model of student burnout has been widely accepted by psychological researchers. Schaufeli et al. (2002) pointed out that student burnout manifests in students as low sense of achievement, emotional exhaustion, peer indifference, alienation between teachers and students, and other phenomena; the cause of these phenomena is excessive learning. As society evolves, student burnout is a common problem in schools around the world, and students in China are facing this problem as well.

Recently, the China Youth Research Center reported that there is an excessive academic burden in primary school. Numerous problems plague primary education as it is currently, such as excessive learning time, overly challenging learning tasks, and unrealistic learning standards (Xu and Zhang, 2017). Additionally, one study reported that 19.2% of Chinese middle school students may have hidden truancy; such adolescents do go to school, and the education system treats them as students, but their psychological states are not truly focused on school activities (Zhou and Tan, 2012). They are reluctant to learn and refuse challenging learning tasks. They feel isolated in school, do not like it, and have a low awareness of their personal learning abilities, even addicted to the Internet (Gong et al., 2021). A study found that 81.3% of Chinese high school students feel great pressure to study, and compared these students to those in the United States, Japan, and Korea. The study thus reported that Chinese high school students spend the most time studying, with 55.3% of Chinese students usually spending more than 2 h a day on homework, compared to 40.2, 13.1, and 10.8% in the United States, Korea, and Japan, respectively (Zhao et al., 2018).

The education system in mainland China can be divided into four stages: preschool education (kindergarten), primary education (primary school), secondary education (including middle and high school), and higher education (college). One of the most common concerns for parents and students is the Nationwide Unified Examination for Admissions to General Universities and Colleges (referred to as “gaokao,” the college entrance examination), which is held between secondary education and higher education. The college entrance exam is scheduled by the Chinese Ministry of Education and administered by the Examination Center of the Ministry of Education and the provincial examination bureaus. The exam is usually held on the 7 and 8th of June (lasting up to 3 days in some areas). The college entrance exam is a fair screening process for excellent high school students to enter college and further their education. Influenced by Confucianism, most Chinese students aspire to enter university for further study, which creates an increasingly competitive environment. Thus, students have to spend much time studying to obtain better grades; however, when the learning time and tasks of these students exceed their physical and mental capacity, they become prone to burnout, which seriously affects their physical and mental health. Therefore, it is necessary to study student burnout in the Chinese context, as the education systems in Hong Kong, Macau, and Taiwan are different from those in mainland China (Liu et al., 2020). Hence, the focus of this study is on mainland China.

### Resilience and Its Positive Impact on Student Burnout

The term “resilience” originated from child developmental psychology and refers to “a construct representing the maintenance of positive adaptation despite significant adversity” (Infurna and Luthar, 2016). In the 1980's, a survey found that some teenagers, although experiencing long-term adversity, had positive emotions and high levels of ability (Werner and Smith, 1982). Subsequent researchers suggested that some factors moderate the relation between high-risk environments and the expected adverse adaptation of students, thus exploring protective factors in well-adapted individuals. These protective factors are collectively called resilience (Masten et al., 1990). At present, there is no consensus on the definition of resilience in psychological researchers. Luthar et al. (2000) summarized three representative definitions of resilience based on the literature. The first definition of resilience is based on results, holding that resilience should be defined in terms of development outcomes. Rutter (1993) indicated that resilience was the positive adaptation of individuals in high-risk environments. The second definition regards resilience as a trait or ability. Block and Kremen (1996) stated that resilience refers to a personality trait with which an individual can quickly recover from a difficult situation and adapt to changes flexibly. Connor and Davidson (2003) argued that resilience was the ability to cope with negative life events such as stress, frustration, and trauma. The third definition holds that resilience is “a dynamic process encompassing positive adaptation within the context of significant adversity” (Tugade and Fredrickson, 2004). Although the process view and trait view of resilience are still controversial, scholars have gradually realized that resilience is an individual characteristic with both variability and stability that reflects the ability of individuals to actively deal with adversity and recover quickly.

However, Kaufman et al. (1994) found that, while nearly two-thirds of individuals exhibited academic resilience, only 21% of individuals exhibited resilience in the area of social competence, demonstrating that resilience is multidimensional and that functional imbalances in different resilience domains are common during individual development. Liu et al. (2017) explored the resilience process of individual interactions in the socio-ecological environment, arguing that resilience is dynamic and multidimensional; moreover, they revealed the interactive nature of resilience through a three-tiered spherical structure (which includes core, internal, and external resilience). This evidence suggests that researchers need to discuss the specificity of resilience results. During a resilience study, the specific area of study should be stated, and it should be noted that the findings may not necessarily apply to other areas (Luthar et al., 1993, 2000). Therefore, many researchers have explored specific resilience, such as teacher resilience (Beltman et al., 2011) and emotional and behavioral resilience (Bowes et al., 2010), with other scholars also using situational tests to assess resilience (Pangallo et al., 2016).

Currently, there are two different research orientations: situational and academic resilience. Situational resilience is a “stable level of health without negative outcomes during or following potentially harmful circumstances and is the typical trajectory after exposure to a potentially traumatic event” (Infurna and Luthar, 2016, 2017). Infurna and Luthar (2016, 2017) looked at the resilience trajectories of adults by measuring their life satisfaction levels before and after experiencing a major event to calculate rates of resilience. Trajectory analysis is a modern transformation of the classic person-centered resilience strategy, which can evaluate the major components of a resilience model (Masten and Cicchetti, 2016). On the other hand, academic resilience is defined as “the heightened likelihood of success in school and other life accomplishments despite environmental adversities brought about by early traits, conditions, and experiences,” and is usually measured through questionnaires (Wang et al., 1994). Thus, the definition of situational resilience is based on processes and is mostly measured by calculating rates of resilience, whereas the definition of academic resilience is based on traits and is mostly measured through questionnaires. Therefore, there has been no uniform method for measuring resilience, and the focus of measurement has varied due to the different definitions and types of resilience (Windle et al., 2011; Liu et al., 2017).

Some studies have found that academic resilience is strongly associated with student burnout. Students with low academic resilience are more likely to exhibit dissatisfied behavior in school, while students with high academic resilience and strong executive function adapt better to school life and respond positively to negative events (Connell et al., 1994; Dunn et al., 2008; Masten et al., 2012; Motti-Stefanidi, 2019). As an important psychological resource, academic resilience can relieve the emotional exhaustion and improve the mental health of students; thus showing that student burnout can be solved successfully (Rios-Risquez et al., 2018). Therefore, the focus of this study is on academic resilience. However, the strength of the relationship between resilience and student burnout and the possible moderators of this relationship are unclear. Overall, this study attempted to explore the correlation between academic resilience and student burnout through a meta-analysis to provide advice for educators to mitigate student burnout.

### Measuring Tool

#### Student Burnout

The most widely used tool to measure student burnout is the Maslach Burnout Inventory (MBI). Maslach and Jackson (1981) conducted a large number of interviews and case studies on service staff before the MBI was proposed, which included three factors: exhaustion, depersonalization, and reduced personal accomplishment. There were three versions of the scale: the MBI-HSS (MBI Human Services Survey), the MBI-ES (MBI Educators Survey), and the MBI-GS (MBI General Survey). The MBI-GS, in particular, can be applied to all occupations, not just human services and education. On the basis of the MBI, Schaufeli et al. (2002) developed the MBI-Student Survey (MBI-SS) with college students as samples, including three factors: exhaustion, cynicism, and low self-efficacy.

Subsequently, many Chinese scholars have conducted Chinese context revisions of student burnout scales on these bases. For instance, Lian et al. (2005) developed a burnout scale for college students with reference to the MBI, which contained 35 items, including three factors, namely, dejection, misconduct, and reduced personal accomplishment, and adopted a 5-point scale. Similarly, Chen (2007) developed a burnout scale for college students, which has a total of 35 items, including three factors, namely, dejection, avoidance behavior, and reduced personal accomplishment, using a 5-point scale. In the same year, Hu and Dai (2007) developed an academic burnout scale for middle school students, which consists of 24 items, including four factors, namely, emotional exhaustion, low self-efficacy, teacher-student alienation, and physiological exhaustion, and adopts a 5-point scale. Simultaneously, Xue (2008) also developed a student burnout scale for middle school students, which consists of 20 items scored by five points and includes three factors: low self-efficacy, exhaustion, and alienation. Fang et al. (2009) translated the MBI-SS scale and verified the three-factor model to fit the Chinese school context. This scale has 15 items and uses a 7-point scale. Using the MBI-SS as a calibration tool, Wu et al. (2010) developed a student burnout scale for adolescents (from primary to high school students), which contains 16 items and adopts a 5-point scale, including three factors: exhaustion, learning cynicism, and low self-efficacy. Ma (2010) further revised the college student burnout scale based on the framework of Lian et al. (2005). The scale has 25 items, including three factors, namely, misconduct, dejection, and reduced personal accomplishment, and uses a 5-point scale.

#### Resilience

Wangnild and Young (1993) first developed the Resilience Scale (RS) for elderly women who experienced great setbacks. The RS included two factors: personal competence and acceptance of self and life. Clough et al. (2002) developed the Mental Toughness Questionnaire (MTQ48), which includes 48 items, the responses to which are graded on a 5-point Likert-type scale, including six factors: challenge, commitment, confidence (in abilities and interpersonal skills), and control (over life and emotions). Connor and Davidson (2003) considered that the previous resilience scales could not effectively measure the level of mental resilience. Focusing on the improvement of anxiety, depression, and stress response in PTSD patients, they developed the Connor-Davidson Resilience Scale (CD-RISC), including five factors: notions of personal competence, high standards, and tenacity; trust in instincts, tolerance of negative affect, and the strengthening effects of stress; positive acceptance of change and secure relationships; control; spiritual influences. It comprises 25 items rated on a 5-point scale, has been validated in many fields, and has become one of the most widely used scales of resilience. Subsequently, for adults, Friborg et al. (2003) developed the Adult Resilience Scale (RSA), which consists of 45 items, uses a 7-point scale, and contains five factors: personal competence, social competence, family coherence, social support, and personal structure. Afterwards, McGeown et al. (2016) developed the Mental Toughness Scale–Adolescents (MTS-A) using the same conceptual framework as MTQ48, which contains 18 items and uses a 4-point scale, including six factors (which are the same as those used in the MTQ48).

Over the past decade, many Chinese scholars have revised the resilience scale to fit the Chinese context. Yu and Zhang (2007) revised the Chinese version of the CD-RISC, which includes 25 items and uses a 5-point scale. However, they discovered that the three-factor model, which referred to tenacity, strength, and optimism, was more applicable to the Chinese context. Hu and Gan (2008) found no Chinese resilience scale for adolescents; therefore, they developed their own resilience scale for adolescents, referring to Connor and Davidson (2003) and Yu and Zhang (2007). It has 27 items and five factors: goal focus, interpersonal assistance, family support, emotion control, and positive cognition. Afterwards, Sun et al. (2009) revised the Resilience Scale (RS) based on Wangnild and Young (1993), consisting of 25 items rated on a 7-point scale.

### Gender Differences

When facing adversity, studies have found that girls have higher levels of anxiety and depression than boys (Lloyd and Gartrell, 1981; Lindfors et al., 2012) and are more likely to report higher levels of student burnout. One possible general explanation is that girls are less resilient than boys, and higher anxiety in girls could be explained by specific psychosocial profiles, which deserve further investigation (Hojat et al., 1999; Ronka et al., 2014). Another possible but opposite explanation is that there may not be a difference in the level of resilience between boys and girls (Duckworth and Quinn, 2009), only that resilience plays a different protective role possibly because, in the face of daily routine challenges, boys are more likely to use adaptive coping strategies such as distraction and problem-solving, whereas girls are more likely to use maladaptive coping strategies, such as rumination and self-focusing (Tang et al., 2021).

Furthermore, there were also some specific findings and explanations across different cultural contexts. In Nordic countries, student burnout was higher among girls than boys in adolescence (Salmela-Aro and Tynkkynen, 2012) because girls and boys may experience school stressors differently: girls may experience more internalizing symptoms (Moksnes et al., 2010), whereas boys typically show more problem behaviors and externalizing symptoms (Salmela-Aro et al., 2008). In addition, girls respond more negatively to competitive learning conditions, are more exposed to stressful events, and are more vulnerable to negative effects; therefore, girls may suffer more from school burnout (Ge et al., 2001). In Serbia, female medical college students assessed their physical health status and general stress level as worse than males, which led to higher levels of student burnout (Backovic et al., 2012). On the contrary, some studies have not found gender differences in student burnout and resilience (Dyrbye et al., 2010; Yu and Chae, 2020). Therefore, the current study will examine whether there are gender differences in burnout and resilience in the context of Chinese schools.

### The Replicability Crisis in Psychology

Can the results of psychological research be replicated? With the discovery that many published psychological research results cannot be successfully replicated in new samples, this problem has received increasing attention, with researchers finding that many previous studies had results that were overly optimistic and effect sizes that were overestimated (Francis, 2014). Based on this, the Center for Open Science (COS) organization repeated 100 psychological studies (Hartgerink and Pernet, 2015) and found that the summary effect size in repetitive studies was half of that in the original studies. The results in 97% of the original studies were statistically significant, but only 36% of the repetitive studies had significant results. Therefore, researchers began to doubt the credibility of psychological research results and, thus, discussed the “replicability crisis” (Baker, 2016). The replicability crisis is a term used by psychologists in the current introspection phase, and the psychology community is undergoing a revolution (Spellman, 2015).

Biases affect the development of science, and they are the important causes of the replicability crises. However, some researchers suggest that metascience can still rescue replicability crises (Schooler, 2014). The first mode of bias to be discussed is publication bias, i.e., the phenomenon that significant results are more likely to be published. At present, there are many mature and effective tests for publication biases, such as funnel plots and the Egger regression (Song et al., 2013). Fanelli and Ioannidis (2013) reanalysed 82 meta-analyses in softer research and found evidence of a “US effect,” which means that “US studies may overestimate effect sizes in softer research.” Fanelli et al. (2017) explored biases in the entire field of science, including small-study effects, gray literature biases, decline effects, US effects, and so on. The term “small-study effect” refers to the phenomenon of studies that are smaller (of lower precision) reporting effect sizes of larger magnitude, which could be due to selective reporting of results or genuine heterogeneity in the study design that results in larger effects being detected by smaller studies (Sterne et al., 2000). The term “gray literature bias” refers to the phenomenon of studies being less likely to be published if they yielded smaller and/or statistically nonsignificant effects and, therefore, might only be available in doctoral theses, conference proceedings, books, personal communications, and other forms of “gray” literature (Song et al., 2010). The term “decline effect” refers to the phenomenon of decreasing effect sizes in repeated studies over time (Schooler, 2011). The decline effect is an important indicator to explore the replicability crisis. Gong and Jiao (2019) found declining effects in emotional intelligence, showing that the effect sizes in the field of emotional intelligence have decreased with time. Jiao et al. (2020) found that individual self-efficacy increases over time, but its predictive effects decline with time. Therefore, the current study will examine whether there are possible biases in burnout and resilience in the context of Chinese schools.

### The Current Study

Based on the aforementioned theory and research, the aim of the current study is to provide the first meta-analysis of the relationship between resilience and student burnout; similarly, it is also the first meta-analysis on this relation in the context of Chinese schools. Compared with empirical research and narrative reviews, meta-analyses have many advantages, such as being systemic, transparent, and replicable (Borenstein et al., 2011). Currently, scholars are concerned with the limitations of empirical research. On the one hand, there are sampling errors in empirical research, so the samples are not representative. For example, most empirical studies on resilience and student burnout adopt convenient sampling methods, which cannot represent the context of Chinese schools. On the other hand, the significant results of empirical research may be statistical flukes (i.e., repeated studies did not yield significant results), whereas meta-analysis allows us to combine the effects and evaluate the statistical significance of the summary effect (Ioannidis, 2005). For the narrative review, considerable evidence suggests that this research method is subjective and untransparent. For example, researchers may selectively report studies that support their views and ignore results that are contrary to them (John et al., 2012). Concerns such as these suggest the value of the meta-analysis on the relationship between resilience and student burnout in prior research. The meta-analysis, as a research method, can improve the efficiency of statistical analysis, compensate for the sampling errors in empirical studies, and determine universal conclusions and differences among studies; in this way, it can explore the biases in the field of research. Thus, it helps researchers reflect on the shortcomings of prior research and provide suggestions for the direction of subsequent research. Moreover, it is an important method for psychology to face its replicability crisis. Accordingly, the purpose of the current study is to explore whether resilience is related to student burnout in the context of Chinese schools and the changing trend of resilience and student burnout using meta-analysis. Moreover, we will explore gender differences and possible biases, including publication biases, small-study biases, gray literature biases, and decline effects.

## Method

### Criteria for Including and Excluding Studies

A study was included if it met the following criteria. (1) The study focused on the relationship between resilience and student burnout. (2) The study reported sufficient statistical detail to allow the calculation of correlations between resilience and student burnout, such as correlation coefficients (*r*), means (*M*), standard deviations (*SD*), sample sizes (*n*), and so on. (3) The study sample included students from primary school to university in mainland China. (4) The study was conducted from January 1979 to July 2020, as many documents were lost before 1979, and most periodical Chinese databases were established in January 1979 or later.

### Literature Search and Selection

The first author and the corresponding author independently conducted a literature search using computer and manual methods on July 9, 2020, to gather all available relevant studies to date. For literature in English, we searched the PsycINFO, ERIC, ProQuest, Web of Science, and Proquest Theses and Dissertations Global databases and developed the following search terms: (“Resilience” OR “Mental Resilience” OR “Academic Resilience” OR “Psychological Resilience” OR “Flexibility” OR “Psychological Flexibility” OR “Elasticity” OR “Mental Elasticity”) AND (“Burnout” OR “School Burnout” OR “Student Burnout” OR “Learning Burnout” OR “Academic Burnout” OR “Study Burnout” OR “Study Lassitude” OR “Languid Learning” OR “Academic Collapse”). For the Chinese literature, we searched based on the CNKI database, the Database of Chinese Sci-tech Journals (WIP Journals), and the Wanfang Database and developed the following search terms: (“弹性” OR “心理弹性” OR “韧性” OR “心理韧性” OR “复原力” OR “恢复力” OR “心理耐挫力” OR “心理承受力”) AND (“倦怠” OR “学业倦怠” OR “学生倦怠” OR “学习倦怠” OR “学术倦怠” OR “学习厌烦” OR “学业崩溃”).

**Figure 1:**
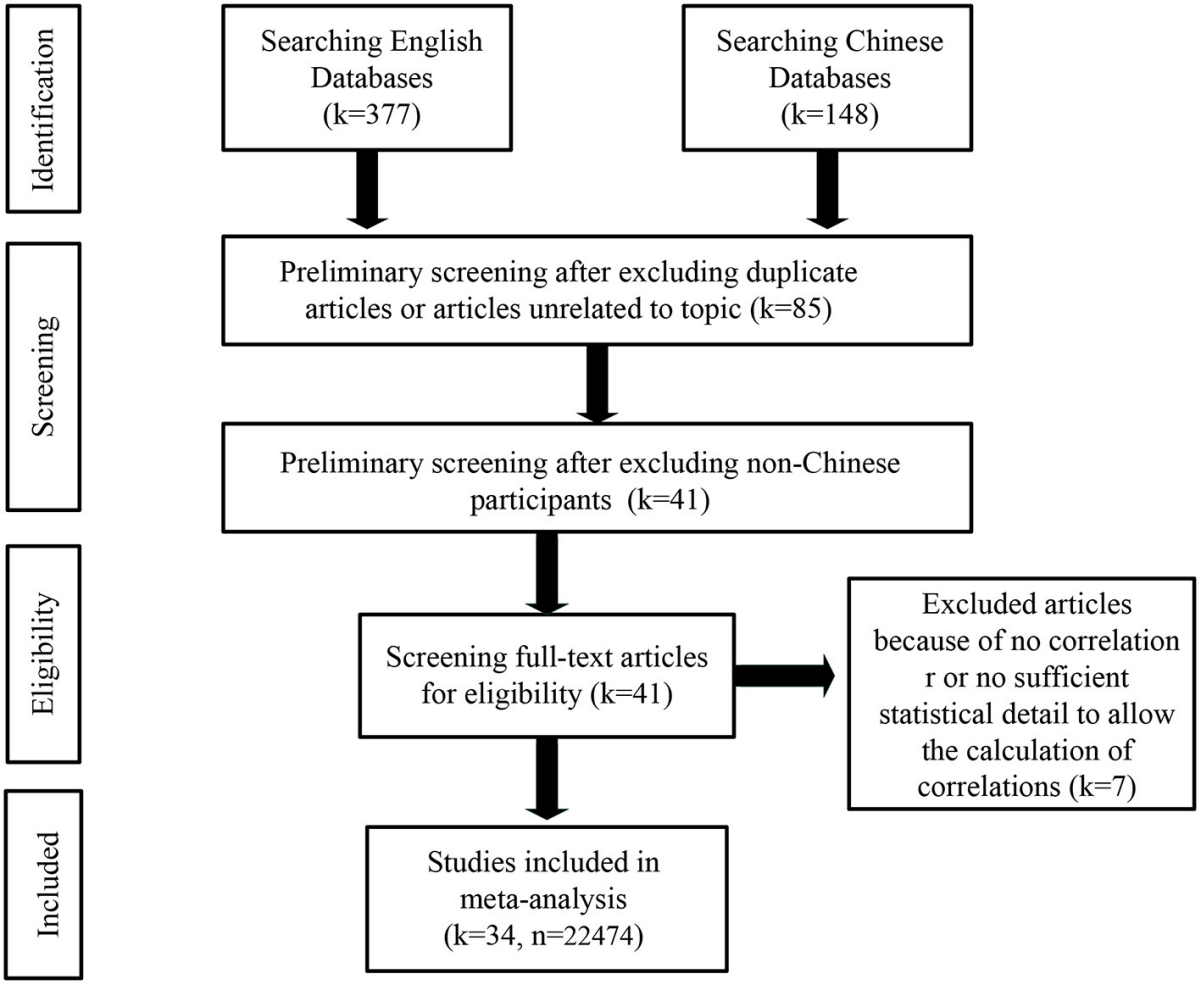
The flow chart of the selection process. *k*, number of articles; *n*, total sample size.

Through the search, we obtained 377 studies from the English databases and 148 studies from the Chinese databases. To ensure that the reliability of this study was robust (referring to Loomes et al., 2017; Nuijten et al., 2017; Gong and Jiao, 2019; Jiao et al., 2020), two independent recorders (i.e., the first author and the corresponding author) double-coded all the collected studies, achieving an intraclass correlation (ICC) of 0.95 for coding comparison. The two independent recorders then discussed and corrected the discrepancies in individual coding. We used the following criteria to address duplicate studies and to exclude studies: (1) Studies that contained obvious errors (e.g., statistical error or data plagiarism) were excluded. (2) If multiple studies were based on the same data, we kept the earliest study and excluded all later studies. (3) If a study was published both in a journal and as a dissertation, we kept the earliest study and excluded the later study. Subsequently, we conducted a preliminary screening to exclude duplicate studies or studies unrelated to the topic, and a total of 440 studies were excluded. We then read the abstracts and excluded 44 more studies because they had non-Chinese participants. For the remaining 41 studies, we screened the full text and excluded another seven studies that did not report a correlation coefficient and could not calculate a correlation coefficient based on the information provided. Following the above selection process (see Figure 1 for the flow chart), 34 studies were included in the meta-analysis (see Table 1 for details). The total sample size was 22,474, and a total of 81 effect sizes were obtained. We divided all the studies into two categories: unpublished journals (master's or doctoral dissertations, coming to a total of 16 studies) and published journals (CSSCI, SSCI, or other journals, coming to a total of 18 studies). All these studies were conducted from 2011 to 2020. With the exception of Zhang and Fan (2015), all studies that reported mean values of resilience used scales ranging from 1 to 5. Therefore, referring to Borenstein et al. (2011), we performed a scale range conversion for the values reported by Zhang and Fan (2015). Additionally, all studies that reported mean values of student burnout used scales ranging from 1 to 5, so no scale range conversion was required.

**Table 1:** Studies included in the meta-analysis (*k* = 34, *n* = 22,474).

**Study**	**Publication type**	**Resilience**				**Student burnout**				**Sample**					**Effect sizes and 95% CI**		
		**Measure tool**	**Range**	***M***	**SD**	**Measure tool**	**Range**	***M***	**SD**	**type**	**Grade level**	***N***	***Mage***	**%Male**	***r***	**LL**	**UL**
Wu (2020)	J	Yu and Zhang (2007)	1–5	—	—	Lian et al. (2005)	1–5	2.91	0.54	C	All	992	18.46 ± 2.01	38.31	−0.247	−0.305	−0.188
Zhao (2019)	D	Hu and Gan (2008)	1–5	3.47	0.64	Wu et al. (2010)	1–5	2.25	0.59	P	5th and 6th	526	—	54.56	−0.646	−0.693	−0.593
Pan (2019)	D	Hu and Gan (2008)	1–5	3.04	0.78	Hu and Dai (2007)	1–5	2.72	0.76	Ju	1th and 2th	254	—	33.46	−0.64	−0.707	−0.561
Gao et al. (2018)	J	Yu and Zhang (2007)	1–5	2.448	0.56	Lian et al. (2005)	1–5	2.821	0.516	C	All	360	19.24 ± 1.38	46.11	−0.452	−0.531	−0.366
Liu et al. (2018)	J	Hu and Gan (2008)	1–5	3.352	0.404	Lian et al. (2005)	1–5	2.928	0.472	C	All	542	20.06 ± 1.54	47.97	−0.47	−0.533	−0.402
Tan and Huang (2017)	J	Yu and Zhang (2007)	1–5	3.4	0.4	Chen (2007)	1–5	2.3	0.6	C	All	61	—	49.18	−0.47	−0.645	−0.247
Zhang (2016)	D	Hu and Gan (2008)	1–5	3.44	0.56	Hu and Dai (2007)	1–5	2.35	0.65	Ju	All	876	—	43.15	−0.61	−0.65	−0.567
Li (2016)	D	Hu and Gan (2008)	1–5	—	—	Hu and Dai (2007)	1–5	—	—	Ju	All	549	—	49	−0.569	−0.623	−0.51
Liao (2016)	D	Hu and Gan (2008)	1–5	3.53	0.59	Wu et al. (2010)	1–5	2.47	0.53	Ju	All	654	—	50.15	−0.518	−0.572	−0.46
Wu et al. (2016)	J	Hu and Gan (2008)	1–5	—	—	Hu and Dai (2007)	1–5	2.6	0.68	H	All	676	—	39.79	−0.497	−0.552	−0.438
Zhang and Fan (2015)	J	Friborg et al. (2003)	1–7	5.02^*^	0.86^*^	Lian et al. (2005)	1–5	2.76	0.728	H	1th and 2th	180	—	100	−0.29	−0.419	−0.15
Wang et al. (2015)	J	Yu and Zhang (2007)	1–5	—	—	Lian et al. (2005)	1–5	2.79	0.4	C	All	197	—	54.31	−0.239	−0.367	−0.103
Li (2015)	J	Hu and Gan (2008)	1–5	3.306	0.502	Wu et al. (2010)	1–5	2.923	0.671	H	All	229	—	35.37	−0.581	−0.661	−0.488
Chen (2014)	D	Hu and Gan (2008)	1–5	3.24	0.47	Hu and Dai (2007)	1–5	2.82	0.55	H	3th	509	—	49.71	−0.485	−0.549	−0.416
Chen (2012)	D	Hu and Gan (2008)	1–5	3.51	0.57	Xue (2008)	1–5	2.67	0.64	Ju & H	Ju. 1th, 2th and H. 1th, 2th	498	—	46.18	−0.65	−0.698	−0.596
Zhang et al. (2019)	J	Hu and Gan (2008)	1–5	3.25	0.58	Lian et al. (2005)	1–5	2.766	0.67	H	All	187	—	15.51	−0.788	−0.837	−0.727
Yin (2019)	D	McGeown et al. (2016)	1–4	—	—	Hu and Dai (2007)	1–5	—	—	H	1th and 2th	738	—	43.9	−0.323	−0.386	−0.257
Chen (2016a)	J	Yu and Zhang (2007)	1–5	—	—	Fang et al. (2009)	1–7	—	—	C	All	731	—	56.63	−0.309	−0.373	−0.242
Chen (2016b)	J	Yu and Zhang (2007)	1–5	—	—	Fang et al. (2009)	1–7	—	—	C	All	487	—	56.67	−0.308	−0.386	−0.225
Zhao et al. (2015)	J	Hu and Gan (2008)	1–5	—	—	Hu and Dai (2007)	1–5	—	—	H	All	674	—	50	−0.497	−0.552	−0.438
Hu (2015)	D	Yu and Zhang (2007)	1–5	3.608	0.546	Lian et al. (2005)	1–5	2.823	0.434	C	All	483	—	80.12	−0.238	−0.32	−0.152
Guo (2014)	D	Hu and Gan (2008)	1–5	3.53	0.421	Lian et al. (2005)	1–5	2.806	0.557	C	All	304	—	51.32	−0.488	−0.569	−0.397
Wang and Zhang (2011)	J	Hu and Gan (2008)	1–5	—	—	Xue (2008)	1–5	—	—	Ju	All	524	—	48.85	−0.64	−0.688	−0.586
Chen et al. (2019)	J	Yu and Zhang (2007)	1–5	3.288	0.517	Lian et al. (2005)	1–5	2.817	0.619	C	2th	404	—	4.95	−0.5	−0.57	−0.423
Yang (2018)	D	Hu and Gan (2008)	1–5	3.442	0.562	Wu et al. (2010)	1–5	2.707	0.665	Ju & H	All	305	—	43.61	−0.557	−0.63	−0.474
Li (2019)	D	Hu and Gan (2008)	1–5	—	—	Xue (2008)	1–5	—	—	Ju	All	3862	—	51.09	−0.453	−0.478	−0.428
Wang (2018)	D	Hu and Gan (2008)	1–5	2.956	0.564	Wu et al. (2010)	1–5	2.586	0.681	Ju	All	479	14.58 ± 1.10	43.63	0.466	0.393	0.533
Luo (2017)	D	Yu and Zhang (2007)	1–5	2.327	0.604	Lian et al. (2005)	1–5	1.74	0.571	C	All	639	—	27.39	−0.697	−0.735	−0.655
Ding (2016)	D	Hu and Gan (2008)	1–5	3.7	0.6	Hu and Dai (2007)	1–5	2.25	0.82	Ju & H	All	710	—	40.85	−0.658	−0.698	−0.614
Zhang (2013)	J	Yu and Zhang (2007)	1–5	3.301	0.64	Ma (2010)	1–5	2.987	0.34	C	All	560	21.14 ± 0.91	69.64	−0.422	−0.488	−0.351
Zhang (2019)	J	Hu and Gan (2008)	1–5	—	—	Wu et al. (2010)	1–5	—	—	H	1th and 2th	485	—	59.59	−0.495	−0.559	−0.425
Zhu (2013)	D	Sun et al. (2009)	1–7	—	—	Hu and Dai (2007)	1–5	—	—	H	All	449	—	46.33	−0.448	−0.519	−0.371
Hou et al. (2017)	J	Hu and Gan (2008)	1–5	3.419	0.448	Lian et al. (2005)	1–5	2.835	0.49	C	All	1628	20.80 ± 3.87	55.28	−0.388	−0.429	−0.346
Cheng et al. (2020)	J	Yu and Zhang (2007)	1–5	2.645	0.515	Lian et al. (2005)	1–5	2.744	0.51	C	1th, 2th, and 3th	1722	—	50.17	−0.434	−0.472	−0.395

*N, sample size; LL, low limit; UL, upper limit; J, journal; D, dissertation; P, primary school students; Ju, junior school students; H, high school students; C, college students*.

* *When this value is incorporated into the meta-analysis; its scale range is converted to 1–5*.

### Coding Procedure

The following elements were coded for each included study (see more details in Table 1): (1) author and publication year; (2) publication type (dissertation or journal); (3) type of resilience measurement tool, mean of resilience (*M*_*resillience*_), and its standard deviation (*SD*); (4) type of student burnout measure, mean student burnout (*M*_*burnout*_), and its standard deviation (*SD*); (5) sample type (primary school, junior school, high school, and college); (6) sample size and gender ratio (the percentage of males); (7) correlation coefficient between resilience and student burnout (some studies did not report specific mean and standard deviations of resilience or student burnout, only their correlation coefficients were reported).

### Effect Sizes Conversion

We used the Comprehensive Meta-Analysis Version 3.3 (CMA 3.3) software for the calculation and conversion of effect sizes (for official software information, see www.Meta-Analysis.com). The current study included 34 studies, all of which reported correlation coefficients (*r*), with 22 of these studies also reporting the mean resilience (*M*_*resilience*_) and 25 reporting the mean student burnout (*M*_*burnout*_). Through the CMA 3.3, this study obtained 81 effect sizes, with a total sample size of 22,474. For the meta-regression, different types of effect sizes should be converted to a single type of effect size. In this study, all the correlation effect sizes were the correlation coefficient (*r*). Next, we converted all *r* instances to Fisher *z*-values using the CMA 3.3.

## Results

We used the CMA 3.3 software for the meta-analysis. First, we carried out the heterogeneity analysis and summary effect size calculations. Then, subgroup analyses were carried out to explore the differences in measuring tools and subject types. Because the publication type was a dichotomous variable (dissertation vs. journal), we also used subgroup analyses to test gray literature biases. For gender differences, the variable we chose was the percentage of males, which was a continuous variable; thus, we used meta-regression to test the gender differences. Similarly, publication years and sample sizes were continuous variables, so meta-regression was also used to test decline effects and small-study effects. Finally, fail-safe *N* and Egger regression were used to test for publication bias. The results are as follows.

### Heterogeneity Analysis

In Table 2, *p*-values are <0.001, suggesting that the overall effect sizes in the studies are heterogeneous. Because of the high heterogeneity, we chose a random-effect model instead of a fixed-effect model in the following analyses.

**Table 2:** Heterogeneity analysis.

	***Q***	**df**	***P***	***I*^**2**^**	**τ^**2**^**	**SE**	**Variance**	**τ**
*M_*resilience*_*	6259.595	21	<0.001	99.665	0.155	0.063	0.004	0.393
*M_*burnout*_*	3714.894	24	<0.001	99.354	0.083	0.032	0.001	0.288
*r*	1068.043	33	<0.001	96.910	0.049	0.017	<0.001	0.222

### The Summary Effect Size Calculation

As shown in Table 3, the meta-analysis includes a total of 34 articles, all of which reported correlation coefficients (*r*), with 22 of these studies also reporting the mean resilience (*M*_*resilience*_) and 25 reported the mean student burnout (*M*_*burnout*_). The summary effect size of resilience was 3.263 (*p* < 0.001), the summary effect size of student burnout was 2.655 (*p* < 0.001), and the summary effect size of the relationship between resilience and student burnout was −0.474 (*p* < 0.001). It shows that resilience is negatively correlated with student burnout.

**Table 3:** The summary effect size calculation.

			**Effect size and 95% CI**				**Test of null (2-Tail)**	
**Effect size**	***K***	***n***	**Estimate**	**SE**	**LL**	**UL**	***Z***	***p***
*M_*resilience*_*	22	12,110	3.263	0.084	3.098	3.428	38.797	<0.001
*M_*burnout*_*	25	13,975	2.655	0.058	2.542	2.769	45.872	<0.001
*r*	34	22,474	−0.474	—	−0.531	−0.413	−13.220	<0.001

*k, number of effect sizes; N, sample size*.

### Subgroup Analyses

For moderations on the mean of resilience shown in Table 4, the difference of publication type (dissertation vs. journal) was not significant, *Q*_*bet*_ = 0.472, *df* = 1, *p* = 0.492, which means that the effect sizes of resilience are not related to publication types; thus, there is no evidence of gray literature biases in the resilience field. However, the difference in resilience measuring tools was statistically significant, *Q*_*bet*_ = 17.785, *df* = 2, *p* < 0.001, which means that the effect sizes of resilience are related to measurement types. This indicates that studies using the resilience scale by Hu and Gan (2008) reported a higher mean resilience than studies using the resilience scale by Yu and Zhang (2007). Additionally, the difference of sample types was statistically significant, *Q*_*bet*_ = 6.828, *df* = 2, *p* = 0.033, which means that the summary effect size of resilience tends to decrease with the age stages of the participants.

**Table 4:** Moderations on the means of resilience.

**Groups**	***k***	**Effect size and 95% interval**				**Test of null (2-Tail)**		**Heterogeneity**		
		**Mean**	**SE**	**LL**	**UL**	***Z***	***p***	***Q_***bet***_***	***df***	***p***
**Publication type**										
D	12	3.316	0.112	3.097	3.536	29.638	<0.001	0.472	1	0.492
J	10	3.199	0.129	2.947	3.451	24.885	<0.001			
Overall	22	3.266	0.084	3.100	3.431	38.693	<0.001			
**Type of resilience measure**										
Hu and Gan (2008)	14	3.372	0.045	3.285	3.460	75.313	<0.001	17.785	2	<0.001
Yu and Zhang (2007)	7	3.002	0.186	2.637	3.367	16.121	<0.001			
Friborg et al. (2003)	1	3.590	0.046	3.499	3.681	77.685	<0.001			
Overall	22	3.464	0.032	3.402	3.526	109.312	<0.001			
**Sample type**										
College	10	3.132	0.146	2.846	3.417	21.498	<0.001	6.828	2	0.033
Junior/high	11	3.365	0.068	3.232	3.498	49.551	<0.001			
Primary	1	3.470	0.028	3.415	3.525	124.349	<0.001			
Overall	22	3.445	0.025	3.395	3.495	135.548	<0.001			

*k, number of effect sizes; J, journal; D, dissertation; LL, low limit; UL, upper limit*.

For moderations on the mean student burnout shown in Table 5, the difference in publication types (dissertation vs. journal) was statistically significant, *Q*_*bet*_ = 6.797, *df* = 1, *p* = 0.009, which indicates gray literature biases in the student burnout field. This shows that studies published in journals tend to report higher means of student burnout. However, we should interpret this with great caution, as the limitation of this finding will be discussed later. The difference in student burnout measurement tools was statistically significant, *Q*_*bet*_ = 185.647, *df* = 5, *p* < 0.001, which means that the effect sizes of student burnout are related to measurement type. This indicates that studies using the Chinese version of the student burnout scale prepared by Lian et al. (2005), which is more widely used, tend to report higher means of student burnout. The difference of sample types was statistically significant, *Q*_*bet*_ = 56.983, *df* = 2, *p* < 0.001, which indicates that the summary effect size of student burnout tends to increase with the age stages of the participants.

**Table 5:** Moderations on the means of student burnout.

**Groups**		**Effect size and 95% CI**				**Test of null (2-Tail)**		**Heterogeneity**		
	***k***	**Mean**	**SE**	**LL**	**UL**	***Z***	***p***	***Q_***bet***_***	***df***	***p***
**Publication type**										
D	12	2.516	0.102	2.316	2.715	24.712	<0.001	6.797	1	0.009
J	13	2.794	0.033	2.730	2.859	85.051	<0.001			
Overall	25	2.768	0.031	2.707	2.829	88.530	<0.001			
**Type of student burnout measure**										
Lian et al. (2005)	12	2.728	0.087	2.558	2.898	31.408	<0.001	185.647	5	<0.001
Wu et al. (2010)	5	2.585	0.100	2.389	2.781	25.826	<0.001			
Hu and Dai (2007)	5	2.547	0.111	2.330	2.765	22.984	<0.001			
Ma (2010)	1	2.987	0.014	2.959	3.015	207.898	<0.001			
Xue (2008)	1	2.670	0.029	2.614	2.726	93.099	<0.001			
Chen (2007)	1	2.300	0.077	2.149	2.451	29.939	<0.001			
Overall	25	2.893	0.012	2.869	2.918	234.054	<0.001			
**Sample type**										
College	12	2.710	0.085	2.543	2.878	31.779	<0.001	56.938	2	<0.001
Junior/high	12	2.633	0.058	2.520	2.746	45.673	<0.001			
Primary	1	2.250	0.026	2.200	2.300	87.463	<0.001			
Overall	25	2.342	0.023	2.297	2.386	103.387	<0.001			

*k, number of effect sizes; J, journal; D, dissertation; LL, low limit; UL, upper limit*.

For moderations on the relationship between resilience and student burnout shown in Table 6, the difference in publication types (dissertation vs. journal) was not significant, *Q*_*bet*_ = 0.320, *df* = 1, *p* = 0.571, which means that the relationship between resilience and student burnout is not related to publication types. The difference in sample types was statistically significant, *Q*_*bet*_ = 29.063, *df* = 2, *p* < 0.001, which means that the relationship between resilience and student burnout is related to the grades of the participants. This indicates that the summary effect size of the relationship between resilience and student burnout tends to decrease with the age stages of the participants. The difference in resilience measuring tools was statistically significant, *Q*_*bet*_ = 18.501, *df* = 4, *p* < 0.001, which means that the relationship between resilience and student burnout is related to resilience measure types. This indicates that studies using the resilience scale by Hu and Gan (2008) reported a higher correlation coefficient than studies using the resilience scale by Yu and Zhang (2007). The difference in student burnout measuring tools was statistically significant, *Q*_*bet*_ = 31.786, *df* = 6, *p* < 0.001, which means that the relationship between resilience and student burnout is related to student burnout measure types; see more details in Table 6.

**Table 6:** Moderations on the relationship between resilience and student burnout.

**Groups**		**Effect size and 95% CI**			**Test of null (2-Tail)**		**Heterogeneity**		
	***k***	***r***	**LL**	**UL**	***Z***	***p***	***Q_***bet***_***	***df***	***p***
**Publication type**									
D	16	−0.492	−0.590	−0.379	−7.572	<0.001	0.320	1	0.571
J	18	−0.457	−0.512	−0.398	−13.352	<0.001			
Overall	34	−0.464	−0.513	−0.412	−15.339	<0.001			
**Sample type**									
College	14	−0.412	−0.480	−0.339	−10.078	<0.001	29.063	2	<0.001
Junior/high	19	−0.507	−0.589	−0.414	−9.244	<0.001			
Primary	1	−0.646	−0.693	−0.593	−17.573	<0.001			
Overall	34	−0.532	−0.570	−0.493	−21.605	<0.001			
**Type of resilience measure**									
Hu and Gan (2008)	20	−0.526	−0.600	−0.443	−10.543	<0.001	18.501	4	0.001
Yu and Zhang (2007)	11	−0.402	−0.492	−0.304	−7.450	<0.001			
Sun et al. (2009)	1	−0.448	−0.519	−0.371	−10.183	<0.001			
McGeown et al. (2016)	1	−0.323	−0.386	−0.257	−9.082	<0.001			
Friborg et al. (2003)	1	−0.290	−0.419	−0.150	−3.972	<0.001			
Overall	34	−0.397	−0.433	−0.359	−18.711	<0.001			
**Type of student burnout measure**									
Lian et al. (2005)	12	−0.456	−0.541	−0.361	−8.452	<0.001	31.786	6	<0.001
Hu and Dai (2007)	9	−0.531	−0.598	−0.457	−11.811	<0.001			
Wu et al. (2010)	6	−0.418	−0.684	−0.053	−2.227	0.026			
Xue (2008)	3	−0.586	−0.710	−0.426	−6.081	<0.001			
Fang et al. (2009)	2	−0.309	−0.359	−0.257	−11.106	<0.001			
Chen (2007)	1	−0.470	−0.645	−0.247	−3.885	<0.001			
Ma (2010)	1	−0.422	−0.488	−0.351	−10.623	<0.001			
Overall	34	−0.400	−0.432	−0.368	−21.734	<0.001			

*k, number of effect sizes; J, journal; D, dissertation; LL, low limit; UL, upper limit*.

### Meta-Regression

As shown in Table 7, the relationships between effect size and sample size were not significant, *p* > 0.05, so we found no evidence of small-study effects.

**Table 7:** Mixed-effects regression (maximum likelihood).

	**B**	**SE**	**LL**	**UL**	***Z***	***p***	***Q_***model***_***	***df***	***P***	***R^**2**^***
**Moderations on** ***M**_***resilience***_*										
Time	−0.070	0.034	−0.137	−0.003	−2.044	0.041	4.180	1	0.041	0.160
*%Male*	0.623	0.378	−0.117	1.363	1.649	0.099	2.719	1	0.099	0.110
*n*	<0.001	<0.001	−0.001	<0.001	−0.885	0.376	0.784	1	0.376	—
**Moderations on** ***M**_***burnout***_*										
Time	−0.014	0.027	−0.067	0.039	−0.523	0.601	0.273	1	0.601	—
*%Male*	0.291	0.304	−0.304	0.887	0.958	0.338	0.918	1	0.338	—
*n*	<0.001	<0.001	<0.001	<0.001	−0.058	0.954	0.003	1	0.954	—
**Moderations on** ***r***										
Time	0.010	0.020	−0.028	0.048	0.518	0.604	0.268	1	0.604	—
*%Male*	0.608	0.261	0.095	1.120	2.325	0.020	5.407	1	0.020	0.139
*n*	<0.001	<0.001	<0.001	<0.001	0.400	0.689	0.160	1	0.689	—

*Time, Publication year; %Male, Percentage of males; N, sample size*.

**Figure 2:**
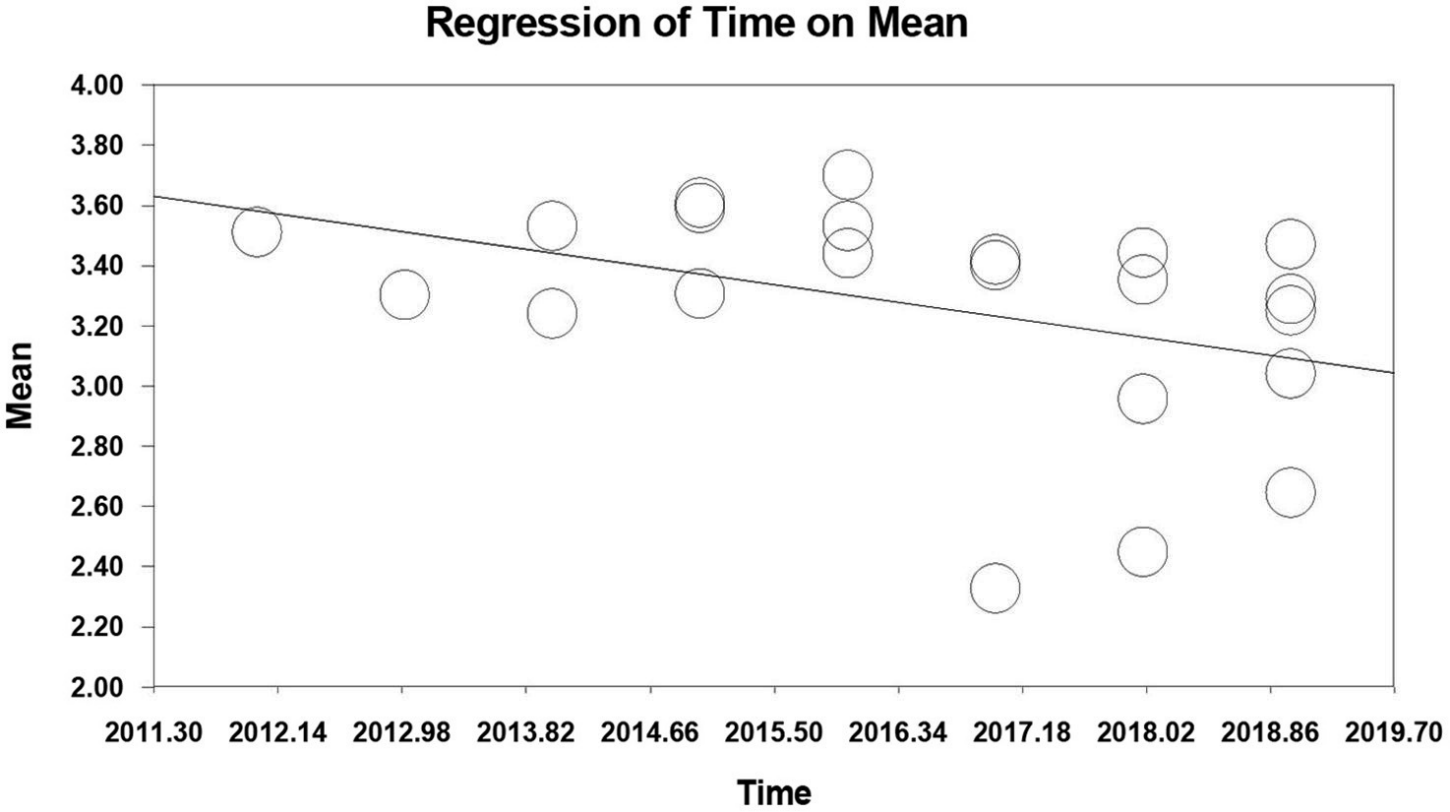
Meta-regression of time on resilience.

For decline effects, the relationship between resilience and publication year was stronger than we would expect by chance, *Q*_model_ = 4.18, *df* = 1, *p* = 0.041. This shows that, with the publication year included in the model, the between-study variance can be explained. The slope was also significantly less than zero, *Z* = −2.044, *p* = 0.041. The *R*^2^ of the regression is 0.160, which is regarded as the magnitude of the effect of the publication year. This indicates that the year of publication can explain 16% of the changes in the effect sizes of resilience. We plot the relationship between resilience and publication year in Figure 2. This illustrates that the summary effect size of resilience decreases significantly with time and that there are declining effects in the resilience field. However, the relationship between student burnout and publication year was not significant, *p* > 0.05, indicating that the measurement of student burnout was relatively stable. The relationship between the correlation coefficient and publication year was also not significant, *p* > 0.05, which shows that the relationship between resilience and student burnout in Chinese students was relatively stable.

**Figure 3:**
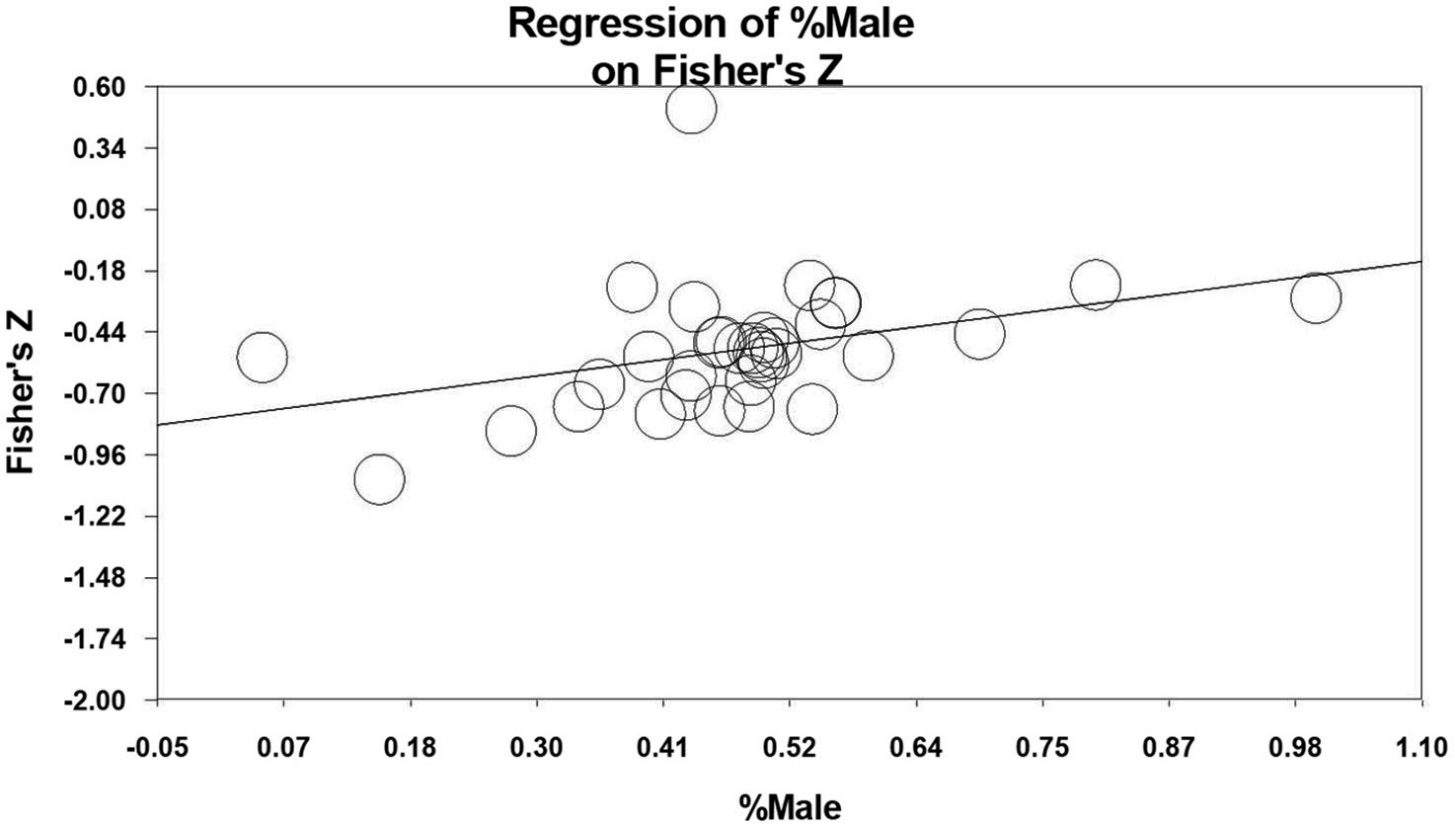
Meta-regression of the percentage of males on the relationship between resilience and student burnout.

Regarding gender differences, the relationship between resilience and the percentage of males was marginally significant, *Q*_model_ = 2.719, *df* = 1, *p* = 0.099. The slope is also marginally significant and more than zero, *Z* = 1.649, *p* = 0.099. This indicates that males may have higher psychological resilience. The relationship between student burnout and the percentage of males was not significant, *p* > 0.05, indicating that there was no gender difference in student burnout. However, the relationship between the correlation coefficient and the percentage of males was stronger than we would expect by chance, *Q*_model_ = 5.407, *df* = 1, *p* = 0.02. This shows that, with the percentage of males included in the model, the between-study variance can be explained. The slope was also significantly more than zero, *Z* = 2.325, *p* = 0.02. This illustrates that the relationship between resilience and student burnout increases significantly with the percentage of males. The relationship between resilience and student burnout tends to be higher in the male group. The *R*^2^ of the regression is 0.139, which is regarded as the magnitude of the effect of gender differences. This indicates that the percentage of males can explain 13.9% of the changes in the relationship between resilience and student burnout. In addition, we plot this observation in Figure 3.

### Publication Bias

**Figure 4:**
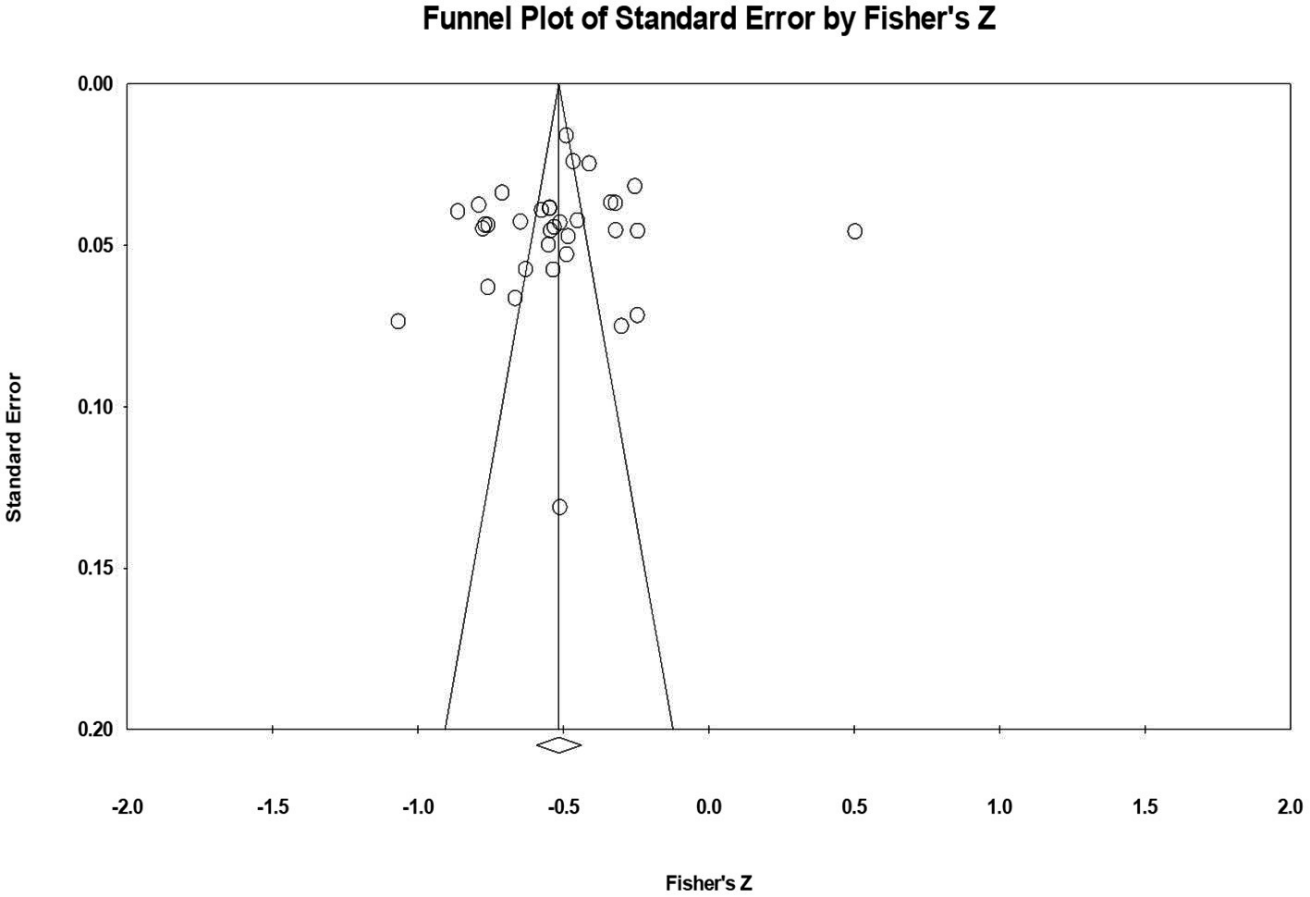
Funnel plot of standard error by Fisher's *Z*.

A Fail-safe *N* is a measure of tolerance for unpublished null findings, i.e., how many unpublished nonsignificant results are required to reverse a significant finding. The Fail-safe *N* for the overall studies in this analysis is 4,027. To consider the relationship between the magnitude and precision of the more informative effect sizes, we performed Egger regression. The Egger regression intercept is −1.583, SE = 2.668, 95% CI = [−7.018, 3.852], *t* = 0.593, *df* = 32, *p* > 0.05, which indicates that there is little likelihood of publication bias in the relationship between resilience and student burnout research. We then plotted a funnel plot of standard error by the Fisher z value as shown in Figure 4. The figure shows that the studies were distributed symmetrically about the mean effect size; hence, the sampling error was random, and publication bias was absent. Therefore, the results of this study are not affected by publication bias.

## Discussion

The aim of the current study was to conduct the first meta-analysis of the relationship between resilience and student burnout; similarly, it is also the first meta-analysis on this relation in the context of Chinese schools. Consistent with our hypotheses, our findings reveal that resilience is negatively related to student burnout in the Chinese context. There was no gender difference in student burnout, regardless of whether male or female students were in China, and the student burnout level was high, which may be due to the influence of an increasingly fierce competitive environment. However, there may be a gender difference in resilience level, which shows that the resilience of male students is higher than that of female students. Publication biases and small-study effects were not found in this study, and we only found evidence of gray literature bias in student burnout; that is, journal studies report higher levels of student burnout than dissertations. A possible reason for this could be that the journal studies overestimated the level of burnout among Chinese students. However, we should interpret this finding with great caution. The limitation of this finding is that this study includes only resilience-related student burnout studies and not all student burnout studies. Thus, this study found a gray literature bias in student burnout; however, this is only a speculation that needs to be verified by subsequent studies.

Another important finding of this study is that, based on the effect sizes of resilience decreasing significantly with publication year, there are declining effects of resilience. Protzko and Schooler (2015) divided decline effects into four types: false-positive decline effects, inflated decline effects, underspecified decline effects, and genuinely decreasing decline effects. The term “false-positive decline effect” describes the circumstance when the effect sizes of subsequent studies decrease over time because there is no true effect, i.e., significant results in the original studies originated from errors in the statistics or methods. The term “inflated decline effect” describes when the effect sizes of subsequent studies decrease over time because the original studies overestimated effect sizes. The term “underspecified decline” effect describes a situation where the effect sizes of subsequent studies decrease over time because a necessary condition in the original studies was underspecified. For example, in the economic game of intuition promoting cooperation, the original study did not report that all the participants were new to the game, which led to a decline in the effect sizes of subsequent studies (Rand et al., 2012). Finally, the term “genuinely decreasing decline effect” describes a circumstance where the effect sizes of subsequent studies decrease over time due to social developments. For instance, with cultural development, the prejudice of white students against African Americans decreased (Dovidio and Gaertner, 1986). Because the summary effect size of resilience is significant, this decline effect is not a false-positive decline effect. Second, all studies included in this meta-analysis used self-report scales to measure resilience, so there are no unclear experimental conditions. Additionally, for resilience, no small-study effect, gray literature bias, or publication bias was found, so the possibility of overestimation or other artificial statistical errors is less likely.

However, in the past few decades, the world has experienced tremendous socioeconomic changes, such as economic growth, urbanization, technological progress, and social change, which have changed not only the way we live but also our culture and psychology. Psychologists have conducted many studies examining cultural and psychological changes, especially in the last two decades. Increasingly more young people choose to live alone, the marriage rate is decreasing, and the divorce rate is increasing; family size is shrinking as well, and the prevalence of different generations living together is declining. These changes have led to changes in personality traits, with individualism-related attributes showing an upward trend, including self-esteem (Twenge et al., 2017), extraversion, conscientiousness, masculinization of women, self-focus, assertiveness self-evaluation (Zhang et al., 2017), and the need for uniqueness (Cai et al., 2018). Some studies have discovered general trends of rising individualism and decreasing collectivism (Cai et al., 2019). Collectivism emphasizes relatedness, in-group (e.g., family or religious organization) control and conformity, harmony, duty, and social hierarchy, whereas individualism emphasizes individual autonomy, freedom, uniqueness, privacy, achievement, and equal opportunity. With the developments in the social culture, people become more focused on themselves as collective behaviors are declining, subsequently leading to the decline of the ability of people to adapt to the collective and their environment, which may be the reason for the decline of resilience over time. Therefore, the current study suggests that this decline effect of resilience with time may be a genuinely decreasing decline effect.

We also found similar decline effects at the individual level; that is, subgroup analysis shows that resilience may decrease with individual age stage because the resilience of the middle school group is lower than that of the primary school group and the college group is lower than that of the middle school group. However, student burnout indicates the opposite, showing an upward trend with age groups. The discovery of age group differences in resilience is consistent with the results of a tracking study in China. Wang and Wang (2018) conducted a 2-year longitudinal study on the resilience and defense mechanisms of Chinese high school students and found that resilience decreased in the total score, family support and interpersonal support dimensions, which may be due to the increased use of immature defense mechanisms by high school students under learning pressure. Since the collective adaptability of students is declining, social adaptability training and training should be increased in school, teachers should actively guide these students to use mature defense mechanisms in the face of difficulties, and parents should provide family support, which is conducive to improving resilience and may help in reducing burnout symptoms.

The following are the limitations of this study and suggestions for future research. First, the studies included in the meta-analysis measured resilience and student burnout in the same way, using questionnaires for both constructs, which could lead to common method biases. However, we could not carry out the common method bias test since we did not obtain all the raw data of the studies. Second, resilience can be measured in multiple ways; for example, trait-based academic resilience is mostly measured by self-report scales, whereas process-based situational resilience is mostly measured by calculating rates of resilience *via* growth curve modeling or growth mixture modeling (Infurna and Luthar, 2016). However, many researchers are beginning to question the reliability of the self-report scale, and studies have found that the level of intelligence measured by the self-report scale was not related to the actual level of intelligence (Paulhus, 2010). Furthermore, some individuals harbor higher social expectations for themselves, thus registering higher scores on resilience scales even if these are not accurate reflections of what they truly felt. The self-report scale has difficulty obtaining accurate scores due to its inability to reveal cover-up behaviors. Therefore, researchers in the field of academic resilience should improve the measurement with reference to more objective measures of resilience, such as situational resilience.

Third, the trait-based approach to defining academic resilience has been questioned because the main contributing factors to the resilience of an individual may vary between people. Recently, studies on the relationship between personality traits and resilience have shown some unexpected findings. Goodman et al. (2017) examined how personality strengths (hope, grit, meaning in life, curiosity, gratitude, control beliefs, and use of strengths) prospectively predict reactions to negative life events, and the results from lagged analyses found that only hope emerged as a resilience factor, which was inconsistent with previous studies. One possible explanation is that, as society changes, the factors that best describe resilience have also changed, which is consistent with the decline effects in the resilience field found in this study. Thus, the definition of resilience may need to be revised over time. Fourth, few longitudinal studies were available to evaluate changes in the impact of resilience on student burnout over time. The use of concurrent methodologies and analyses, which is the norm in psychology, often leads to erroneous conclusions (Goodman et al., 2017). Therefore, we can only assume that there is a correlation, not a causal relationship, between resilience and academic burnout. Follow-up studies should focus on the long-term benefits of resilience for student burnout. Fifth, as the education systems in Hong Kong, Macau, and Taiwan are different from those in mainland China, the focus of this study is on mainland China. Subsequent studies could specifically analyse whether consistent results also exist in Hong Kong, Macau, and Taiwan. Furthermore, it would be worth researching whether these findings would hold in other cultural contexts.

## Conclusion

The current study is the first meta-analysis of the relationship between resilience and student burnout in the context of Chinese schools, and our findings revealed that resilience is negatively related to student burnout in the Chinese context. This study found that there is no gender difference in student burnout, although there may be a gender difference in resilience level, with the findings showing that the resilience of male students is higher than that of female students. Publication biases and small-study effects were not found in this study, and we only found evidence of gray literature bias in student burnout; however, this should be interpreted with great caution because this study only included resilience-related student burnout studies and not all student burnout studies. Thus, there is a gray literature bias in student burnout; however, this is only a speculation that needs to be verified by subsequent studies. Moreover, based on the effect sizes of resilience decreasing significantly with publication year, we found that there may be declining effects on resilience. This decline effect of resilience with time may be a genuinely decreasing decline effect, possibly because, as societies and cultures evolve, people become more focused on themselves; thus, collective behaviors decline, leading to a decrease in the ability to adapt to the collective and the environment. We also found similar decline effects at the individual level; that is, subgroup analysis showed that resilience may decrease with individual age stages, which may be related to the use of immature defense mechanisms against stress by students. Therefore, we suggest that, with the growth of children and the increase in academic pressure, parents should pay more attention to the psychological state of adolescents, actively guide them to use mature defense mechanisms in the face of difficulties, and provide family support, which is conducive to improving resilience and may help in reducing burnout symptoms.

## Data Availability Statement

The original contributions presented in the study are included in the article/supplementary material, further inquiries can be directed to the corresponding author/s.

## Author Contributions

ZG and XJ designed, performed, analyzed the research, and wrote up the research. ZG and QQ critically reviewed and edited the manuscript. CL made substantial revisions. All authors contributed to the article and approved the submitted version.

## Conflict of Interest

The authors declare that the research was conducted in the absence of any commercial or financial relationships that could be construed as a potential conflict of interest.

## Publisher's Note

All claims expressed in this article are solely those of the authors and do not necessarily represent those of their affiliated organizations, or those of the publisher, the editors and the reviewers. Any product that may be evaluated in this article, or claim that may be made by its manufacturer, is not guaranteed or endorsed by the publisher.
